# Dosisanpassung von Upadacitinib bei mittelschwerer bis schwerer atopischer Dermatitis: Erkenntnisse aus einer multizentrischen Studie unter realen Bedingungen

**DOI:** 10.1111/ddg.15908_g

**Published:** 2025-12-11

**Authors:** Flavia Manzo Margiotta, Valentina Dini, Laura Calabrese, Laura Lazzeri, Sofia Lo Conte, Manfredi Magliulo, Giulio Montesi, Leonardo Pescitelli, Nicola Milanesi, Carlo Mazzatenta, Emiliano Antiga, Pietro Rubegni, Massimo Gola, Marco Romanelli, Alessandra Cartocci

**Affiliations:** ^1^ Department of Dermatology University of Pisa Pisa Italy; ^2^ Health Science Interdisciplinary Center Sant'Anna School of Advanced Studies Pisa Italy; ^3^ Dermatology Unit Department of Medical Sciences Surgery and Neurosciences University of Siena Siena Italy; ^4^ Unit of Diagnostic and Therapeutic Neuroradiology Department of Neurology and Human Movement Sciences Azienda Ospedaliero Universitaria Siena Italy; ^5^ Allergological and Pediatric Dermatology Unit Department of Health Sciences University of Florence Florence Italy; ^6^ Unit of Dermatology San Giuseppe Hospital Empoli Florence Italy; ^7^ Department of Dermatology San Jacopo Hospital Pistoia Italy; ^8^ Unit of Dermatology Azienda USL Toscana Nord Ovest Lucca Italy; ^9^ Department of Health Sciences Section of Dermatology University of Florence Florence Italy

**Keywords:** atopische Dermatitis, Dosis, JAK‐Inhibitoren, Upadacitinib, Wirksamkeit, atopic dermatitis, dose, efficacy, JAK inhibitors, Upadacitinib

Sehr geehrte Damen und Herren,

In den letzten Jahren haben sich biologische Therapien und niedermolekulare Wirkstoffe, insbesondere Januskinase (JAK)‐Inhibitoren, als wirksame Medikamente für Patienten mit mittelschwerer bis schwerer atopischer Dermatitis (AD) herausgestellt.^1^ Sie bieten eine schnelle und gezielte Linderung der Erkrankung, die durch chronische Entzündung, starken Juckreiz und erhebliche Beeinträchtigung der Lebensqualität gekennzeichnet ist. Unter den JAK‐Inhibitoren verringerte Upadacitinib – ein oraler selektiver JAK1‐Inhibitor – in mehreren klinischen Studien signifikant den Schweregrad der Erkrankung, die Juckreizintensität und die Schlafstörungen.^2^ Sowohl Tagesdosen von 15 mg als auch 30 mg erzielen deutliche Verbesserungen und bieten Ärzten die Flexibilität, die Behandlung nach dem Schweregrad der Erkrankung und patientenspezifischen Faktoren individuell anzupassen. Diese Flexibilität bei der Dosierung ist besonders wichtig in der klinischen Praxis, wo die Patientenprofile oft von denen in randomisierten kontrollierten Studien abweichen. Die Möglichkeit, die Dosierung nach Ansprechen und Verträglichkeit zu variieren, ermöglicht die Anpassung an klinische Schwankungen im Laufe der Zeit.^3^ Trotz zunehmender Anwendung von Upadacitinib sind Daten aus der Praxis hinsichtlich des Zeitpunkts, der Gründe und der Ergebnisse von Dosisanpassungen begrenzt. Hier könnte ein strukturierter und evidenzbasierter Ansatz für Dosierungsänderungen – Erhöhung oder Verringerung – die personalisierte Behandlungsplanung und das langfristige Krankheitsmanagement erheblich verbessern. Um diese Lücke zu schließen, war das Ziel unserer Studie, Dosisanpassungen von Upadacitinib bei erwachsenen Patienten mit AD zu evaluieren. Die Studie wurde in sechs dermatologischen Zentren in der Toskana, Italien, durchgeführt und umfasste Patienten, die von Mai 2022 bis März 2025 mit Upadacitinib behandelt wurden. Statistische Analysen, darunter Chi‐Quadrat‐Tests, Mann‐Whitney‐U‐Tests oder Student's t‐Tests und Kaplan‐Meier‐Überlebensanalysen, wurden mit R Version 4.3.2 durchgeführt, um Zusammenhänge zwischen Dosis, klinischen Variablen und Medikationsdauer zu untersuchen. Die Patienten willigten schriftlich in die Veröffentlichung ihrer Falldaten ein. Die Studie wurde am 16. Mai 2022 vom *Comitato Etico Regionale per la Sperimentazione Clinica della Toscana* – AREA VASTA SUD EST (Protokollnummer 22045) geprüft und genehmigt.

Insgesamt wurden 58 Patienten, 31 Männer (53,4%) und 27 Frauen (46,6%) mit einem Durchschnittsalter von 32,3 ± 12,7 Jahren, eingeschlossen. Patienten über 65 Jahre waren nicht beteiligt. Von den eingeschlossenen Patienten begannen 40/58 (69,0%) die Behandlung mit der 15‐mg‐Dosis, während 18/58 (31,0%) mit 30 mg begannen. Ausgangsvergleiche ergaben, dass Patienten, die die 30‐mg‐Dosis erhielten, signifikant höhere EASI‐Werte (*Eczema Area and Severity Index*) aufwiesen (23,33 gegenüber 18,08, p  =   0,038), was auf schwerere Erkrankungen hindeutet. Demografische Variablen, Familienanamnese, Krankheitsbeginn oder Verteilung der AD‐Phänotypen – wie klassische Flexural‐, psoriasiforme, diffuse, Handekzeme oder erythrodermische Typen – waren nicht signifikant unterschiedlich, mit Ausnahme einer stärkeren Beteiligung des Halses bei den Patienten, die mit 15 mg begannen (p  =   0,044). Während des Studienzeitraums wurden insgesamt 24 Dosisanpassungen verzeichnet (Tabelle 1). Diese waren bei Patienten, die mit 30 mg behandelt wurden, deutlich häufiger (insgesamt 29: 18 Erstverschreibungen plus 11 Dosiserhöhungen), wobei 13/29 (44,8%) eine Dosisänderung benötigten, verglichen mit nur 11/53 (20,8%) der Patienten, die 15 mg erhielten (insgesamt 53: 40 Erstverschreibungen plus 13 Dosisreduktionen). Die Hauptgründe für Dosisreduktionen bei 30 mg waren unerwünschte Ereignisse (UE), die bei 9/29 (31%) Patienten auftraten, und das Erreichen einer minimalen Krankheitsaktivität (MDA) bei 5/29 (17,2%) Patienten, definiert nach Silverberg et al.^4^ Die 9 gemeldeten AE traten nach durchschnittlich 13,1 ± 8,5 Wochen auf und waren 4/9 akneiforme Reaktionen (44,4%), 3/9 Hypercholesterinämie/Hyperlipidämien (33,3%), 2/9 Neutropenie/Leukopenien (22,2%) und 1/9 Asthenie (11,1%). Unter den Patienten mit unerwünschten Ereignissen und Dosisreduktion hatten 77,8% bereits die MDA erreicht, die in 57,1% der Fälle aufrechterhalten wurde. Umgekehrt war der einzige Grund für die Erhöhung der 15‐mg‐Dosis das Nichterreichen der MDA in 11/53 Fällen (21,2%). Diese Patienten erhielten nach durchschnittlich 34,8 ± 26,5 Wochen eine Dosiserhöhung auf 30 mg und erreichten in 40% der Fälle die MDA. Die ODS‐Kurven ohne MDA‐Ereignisse sind in Abbildung 1 dargestellt.

**TABELLE 1:** Upadacitinib‐Dosisanpassungen. Zusammenfassung der individuellen Dosisanpassungen bei 20 Patienten. Die Zeitpunkte (Wochen) geben an, wann die Anpassungen vorgenommen wurden. Erhöhungen von 15 mg auf 30 mg wurden aufgrund einer unzureichenden Krankheitskontrolle vorgenommen, während Reduzierungen von 30 mg auf 15 mg in erster Linie aufgrund von unerwünschten Ereignissen oder dem Erreichen einer minimalen Krankheitsaktivität (MDA) erfolgten.

Patient	Alter und Geschlecht	Dosisanpassung	Grund für die Anpassung	Zeitpunkt der Anpassung (Wochen)
1	34, weiblich	15 → 30	MDA nicht erreicht	10
2	28, M	30 →15 → 30	AE: Akne (Dosis reduziert) MDA nicht erreicht (Dosis erhöht)	16; 50
3	46, M	30 → 15	AE: Akne	12
4	19, M	15→ 30	MDA nicht erreicht	64
5	34, F	15 →30 → STOP	MDA sowohl mit 15 als auch mit 30 nicht erreicht	25; 32
6	24, F	15→ 30	MDA nicht erreicht	27
7	23, M	15 → 30	MDA nicht erreicht	72
8	37, F	30 → 15	AE: Akne, Hyperlipidämie	12
9	60, M	30 → 15	MDA erreicht	17
10	30, F	30 → 15	AE: Hypercholesterinämie	13
11	28, M	30 → 15	AE: Asthenie	4
12	56, M	30 → 15	MDA erreicht + AE: Hypercholesterinämie	10
13	20, M	15 → 30 → 15	MDA nicht erreicht (Dosis erhöht) AE: Akne (Dosis reduziert)	10; 19
14	29, F	30 → 15	MDA erreicht	10
15	42, F	15 → 30	MDA nicht erreicht	25
16	20, F	30 → 15 → 30	AE: Leukopenie (Dosis reduziert) MDA nicht erreicht (Dosis erhöht)	8; 103
17	27, M	30 → 15	MDA erreicht	13
18	16, F	15 → 30	MDA nicht erreicht	39
19	48, F	15 →30→ 15	MDA nicht erreicht (Dosis erhöht) AE: Neutropenie (Dosis reduziert)	16; 40
20	26; M	30 → 15→ STOP	MDA erreicht; MDA nicht erreicht und klinische Entscheidung zum Wechsel zu Anti‐IL	16; 29

*Abkürzungen*: AE, unerwünschtes Ereignis; F, weiblich; M, männlich; MDA, minimale Krankheitsaktivität

**ABBILDUNG 1:**
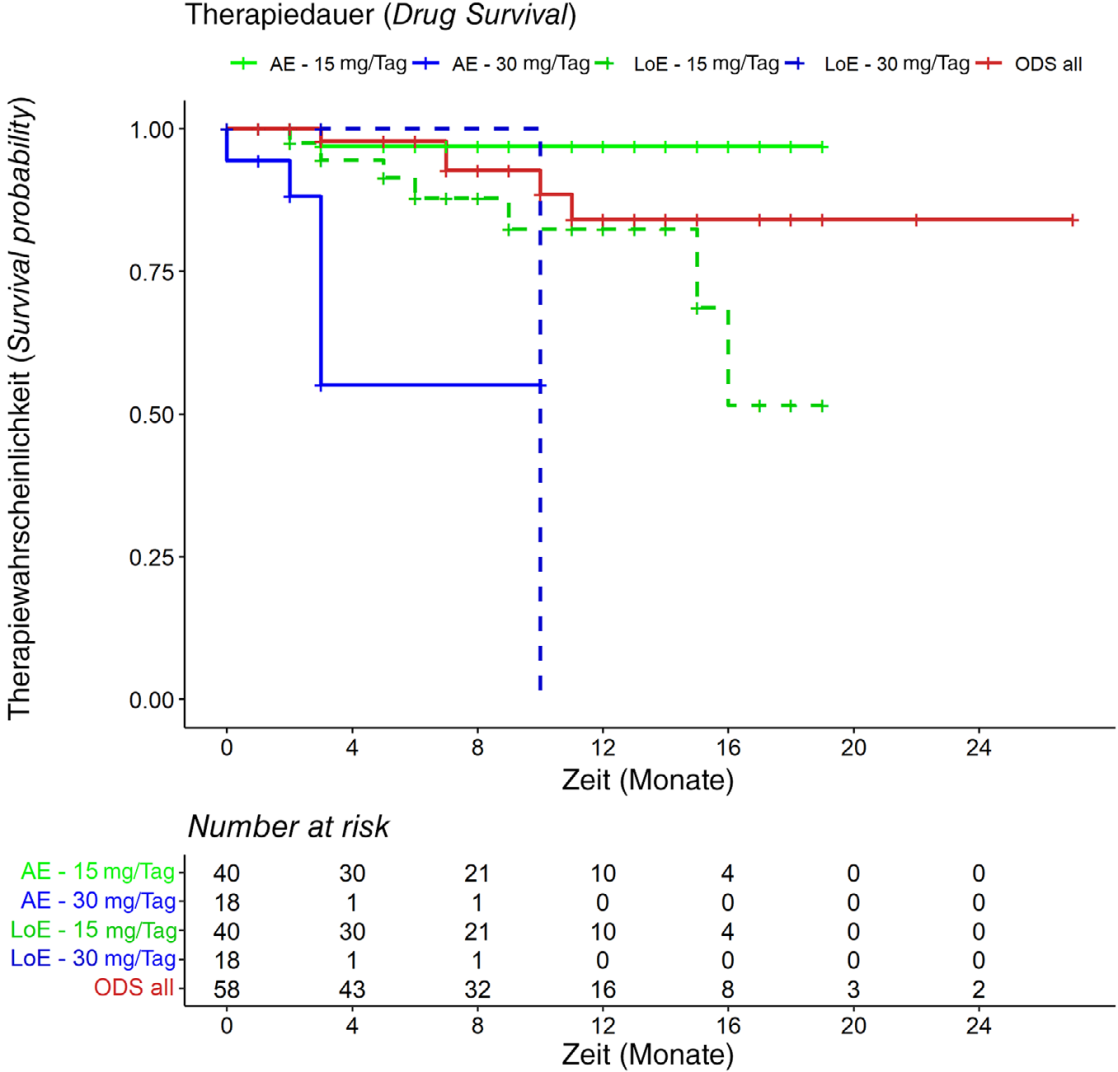
Gesamtmedikamentenüberlebensrate (ODS) verschiedener Dosierungen von Upadacitinib. *Rote Linie*: Gesamtkohorte der mit Upadacitinib behandelten Patienten mit atopischer Dermatitis; *durchgezogene grüne Linie*: Therapiedauer von Upadacitinib 15 mg/Tag in Verbindung mit unerwünschten Ereignissen; gepunktete grüne Linie: 15 mg/Tag in Verbindung mit Wirkungsverlust; durchgezogene blaue Linie: 30 mg/Tag in Verbindung mit unerwünschten Ereignissen; gepunktete blaue Linie: 30 mg/Tag in Verbindung mit Wirkungsverlust. *Abkürzungen*: AE, unerwünschtes Ereignis; LoE, Wirkungsverlust; ODS, Gesamtmedikamentenüberlebensrate

Dies ist die erste Praxisstudie, die klinische Faktoren als Gründe für Dosisänderungen von Upadacitinib bei AD analysiert. Während frühere Studien Wirksamkeitsunterschiede zwischen 15 mg und 30 mg hervorhoben, beschrieb keine Dosisanpassungsprozesse.^5^ Eine kürzlich veröffentlichte Übersichtsarbeit empfahl, Patienten auf der niedrigsten wirksamen JAK‐Inhibitor‐Dosis zu belassen und gleichzeitig individuelle Anpassungen zuzulassen.^6^ Unsere Ergebnisse stützen dieses Modell: 15 mg waren bei etwa 20% der Patienten unzureichend, während jeder fünfte Patient unter 30 mg nach 13,2 Wochen auf 15 mg reduziert werden konnte, wobei in 50% der Fälle eine MDA erreicht wurde. Dieser Zeitrahmen ist kürzer als bei Dupilumab (52 Wochen) und Tralokinumab (16 Wochen).^7^, ^8^ Obwohl Nebenwirkungen unter 30 mg häufiger auftraten, waren sie meist gut behandelbar, insbesondere Akne, eine häufige JAK‐assoziierte Nebenwirkung, die als leicht eingestuft wird.^9^ Zukünftige Studien sollten prädiktive Marker für Nebenwirkungen und das Ansprechen untersuchen, wie dies bereits für Biologika geschehen ist.^10^ Zusammenfassend unterstreichen diese Ergebnisse aus der Praxis die Wirksamkeit, Sicherheit und Flexibilität von Upadacitinib und bestätigen seine Rolle in der personalisierten Behandlung von mittelschwerer bis schwerer AD.

## DANKSAGUNG

Open access Veröffentlichung ermöglicht und organisiert durch Projekt DEAL.

## INTERESSENKONFLIKT

A.E. hat Honorare oder Vergütungen von Abbvie, Almirall, Incyte, Leo Pharma, Lilly, Pfizer und Sanofi erhalten. F.M.M. hat Zahlungen von Abbvie, Almirall, Eli Lilly, Leo Pharma, Pfizer und Sanofi erhalten. M.M. hat Vergütungen von Abbvie und Pfizer erhalten. M.R. hat Unterstützung von Abbvie, Almirall, Convatec, Eli Lilly, Janssen, Leo Pharma, Novartis, Sanofi, UCB und Urgo erhalten. V.D. hat Zahlungen von Abbvie, Almirall, Convatec, Eli Lilly, Janssen, Leo Pharma, Novartis, Pfizer, Sanofi und UCB erhalten.
